# Evaluation of potentially significant drug-drug interactions among patients receiving psychotropic medications: A real-world retrospective study

**DOI:** 10.5339/qmj.2025.107

**Published:** 2025-12-10

**Authors:** Rania Abu-Kuhail, Oraib Abdallah, Mohammed Abu-Hafizah, Shatha Alqam, Yassin Eltorki, Nosyba Ez Eldeen, Sali El Hoseny, Noriya Al-Khuzaei

**Affiliations:** 1 Pharmacy Department, Mental Health Services, Hamad Medical Corporation, Doha, Qatar *Email: rabukuhail@hamad.qa

**Keywords:** Drug interactions, psychotropics, Qatar, mental illness

## Abstract

**Background:**

Drug-drug interactions (DDIs) can lead to adverse events or altered drug effectiveness, and polypharmacy increases the likelihood of DDIs. This study aimed to explore the prevalence of clinically significant DDIs among patients with mental health problems who are prescribed psychotropic medications.

**Methodology:**

This was a retrospective observational study that included patients who had visited the Outpatient Mental Health Pharmacy at Mental Health Services (MHS) in Qatar for medication dispensing. The study covered all individuals over 2 months, totaling 1586 patients. The list of medications was cross-referenced with Lexicomp^®^ to identify potential DDIs. Then, each identified DDI was assessed for risk rating, severity, and reliability rating. Descriptive analysis was applied first. The Shapiro-Wilk test for normality, the chi-square test, the Mann-Whitney *U* test, and binary logistic regression were conducted as appropriate.

**Results:**

Of the 1586 files reviewed, 49% of the patients did not experience any DDIs, while the remaining 51% (*n* = 807) had at least one potential interaction. Among these, 44% had no comorbidities aside from their psychiatric condition. Anxiety disorders (25.65%) and schizophrenia (23.9%) emerged as the most common diagnoses. Patients were found to be taking an average of 4.69 ± 3.36 medications, with 2.23 ± 0.96 of these being psychotropic drugs. On average, each patient experienced 3.15 potential psychotropic DDIs. The majority of the interactions belonged to Category C (86%; *n* = 2192), followed by Category D (7.8%; *n* = 200). The total number of medications was the only statistically significant predictor of having potential “Intervention Required/High-Risk Drug Interactions” (*P* < 0.001).

**Conclusions:**

The study provided valuable insight into the prevalence of potential DDIs among patients prescribed psychotropic medications in Qatar. Notably, the analysis highlighted significant interactions, particularly those associated with severe outcomes, like Torsades de Pointes and anticholinergic intoxication. These findings underscore the need for targeted strategies to mitigate adverse effects and enhance patient safety in clinical practice.

## INTRODUCTION

Mental disorders rank in the top ten causes of morbidity worldwide. The two biggest contributors are major depressive disorder and anxiety disorders. Between 1990 and 2019, the global number of mental disorders increased by approximately 48%, rising from an estimated 654.8 million cases to 970.1 million cases.^1^

Psychotropic drugs (e.g., antidepressants, anxiolytics, stimulants, antipsychotics, mood stabilizers, etc.) are the first-line treatment of mental disorders.^2^ Psychotropic drug usage is increasing worldwide despite its association with serious side effects and drug-drug interactions (DDIs) that may affect the patients’ quality of life, resulting in additional service costs. The off-label use of psychotropic medications, beyond their officially approved indications, has also significantly increased, prompting closer attention.^3–6^ In the United States, at least one in six adults receives a prescription for a psychotropic drug, according to a systematic review done in 2018. High rates of psychotropic drug use have also been found in several vulnerable populations who are more susceptible to the consequences of side effects and DDIs, including children, adolescents, geriatrics, those suffering from dementia, and those with neurodevelopmental disorders like intellectual disabilities and autism.^5^

In 2021, Brauer et al. published a longitudinal study stating that the overall consumption of psychotropic drugs had globally increased in 2019 to reach 34.77 defined daily dose (DDD) per 1000 of population per day from 28.54 DDD per 1000 of population per day in 2008.^2^

Global monitoring studies that compare and track the level of psychotropic drug use have not been done yet, while the small number of studies that have been published internationally about psychotropic drug consumption were restricted to one type of psychotropic drug.^2^

In the state of Qatar, there is a shortage of data regarding psychotropic drug consumption. On the other hand, a few published studies suggest underutilization of some classes of psychotropic drugs.^7,8^

DDIs are defined as a change in a drug’s effect on the body when the drug is taken together with a second drug. Potential DDIs can be pharmacodynamic or pharmacokinetic. Pharmacodynamic interactions occur when two or more drugs are administered together and have either additive or canceling effects on the body. Pharmacokinetic interactions occur when a drug affects the absorption, distribution, metabolism, or excretion characteristics of another drug.^9^ Potential DDIs are known to be the most common cause of adverse drug events, which in turn affect patients’ health in addition to healthcare costs by increasing morbidity, mortality, and hospitalizations^10–13^

DDIs’ global prevalence is of increasing concern, especially in terms of the widespread use of psychotropic medications and the rise of polypharmacy. Studies have been done on psychiatric inpatients, showing that the potential of DDIs among them ranges between 65% and 77%.^14^ Clinically significant DDIs were noticed in patients with schizophrenia spectrum disorders and identified in up to 95%.^14^ One systematic review stated that 42% of psychiatric patients were exposed to at least one QT-prolonging psychotropic interaction, which leads to serious cardiovascular risks.^15^ These interactions are often unrecognized and may lead to adverse outcomes such as serotonin syndrome, neurotoxicity, and increased mortality.^16,17^ Although these risks, documentation, and routine screening of DDIs in psychiatric settings are still inconsistent, particularly in low and middle-income countries.^16,18^

Few studies have emphasized the significance of drug interactions in the psychiatry field, while psychotropic polypharmacy has become one of the major public health problems worldwide.^19^ However, many psychotropic drugs are known to interact with other psychotropic and non-psychotropic co-administered drugs. Common adverse effects related to psychotropic DDIs include increased or decreased effectiveness, neurotoxicity, QT prolongation, serotonin syndrome, and central nervous system depression, as most psychotropic medications cause drowsiness, dizziness, or sedation.^19,20^

Despite that there are many psychotropic medications with DDIs that are clinically insignificant, there are some that can have catastrophic consequences, including the possibility of death.^9^ It may take many weeks for the deadly effect to manifest.^21^

In Qatar, to our knowledge, there was no study focused on identifying DDIs among psychiatric patients receiving medications, and thus, we aim to explore the prevalence, significance, and severity of potential DDIs in patients with mental illness. The primary objective is to determine the prevalence of significant DDIs among patients with psychiatric illness who are utilizing psychotropic medications at Residential Continuous Care Compound 2 - MHS - Hamad Medical Corporation (HMC) in the state of Qatar. Additionally, this study aimed to identify a second objective related to explaining the type, risk rating, severity, onset, and reliability rating of DDIs associated with the treatment of psychiatric illness among psychiatric patients in the State of Qatar.

## METHODOLOGY

### Study setting and sample

This was a retrospective observational study that included patients who had visited the outpatient mental health pharmacy at Residential Community Care Compound under MHS, HMC in Qatar, for medication dispensing. The study covered all individuals with any mental illness on medications for over 2 months, totaling 1586 patients. No exclusion criteria were applied. Through a thorough chart review, researchers identified the active medications of each patient, with a specific focus on those prescribed psychotropic medications. The list of medications was then cross-referenced with Lexicomp to identify potential DDIs. The Lexicomp^®^ Interact risk rating is categorized as “A, B, C, D, or X” as shown in ^22^ The report provides the severity rating, onset, and reliability of each potential DDI based on Lexicomp’s predefined criteria, which is added to the collection sheet outlined in Table 1.

Additionally, demographic information and comorbidity details were gathered to incorporate clinical characteristics into our analysis. The scope of the study exclusively considered and investigated drug interactions involving psychotropic medications. We have excluded patients who were prescribed non-psychotropic medications or those receiving only a single psychotropic medication as monotherapy. We anticipated an average of 40 patients daily seeking medication dispensing, and thus, we aimed to conduct a total population sample over the specified 2-month period (March 1, 2022–May 1, 2022).

### Data collection

A dedicated data collection form was developed to facilitate data collection as outlined in Appendix 1. This form was designed to comprehensively capture all relevant information about the patients, their medications, and potential drug interactions, type of DDI, risk rating, outcome severity, onset of unmanaged interactions, reliability, documented adverse drug reaction outcome, if any. Data collection was conducted systematically, with information entered directly into an Excel sheet at the time of chart review. This approach minimized the risk of transcription errors or misinterpretation of data.

### Data analysis

Statistical Package for Social Sciences, version 29, for data entry and analysis was used. Descriptive statistics were applied first. Frequency and percentage were calculated (mean + SD). Further, we have classified those who had DDI with Categories B, C, D, and X into two groups, labeling them as: Low-to-Moderate Risk (Categories B, C), naming it as “Monitor/Low-Risk Interactions,” and High-Risk (Categories D and X), naming it as “Intervention Required/High-Risk Interactions.” Normality tests were conducted to check the distribution of variables. The Shapiro-Wilk test was of *P*-value ≤ 0.05, the variables were not normally distributed, and thus non-parametric tests were used.

For categorical variables, the chi-square test was used. The Mann-Whitney *U* test was used for continuous data. A *P*-value of ≤0.05 was considered a significant difference. Binary Logistic Regression was used for checking predictors of having a DDI that is with “Intervention Required/High-Risk Interactions.” If the odds ratio (Exp(B)) value was >1, the predictor increases the likelihood of having “Intervention Required/High-Risk Interactions.”

## RESULTS

### Demographics and clinical characteristics

Of the 1586 patient records assessed for those receiving medications from Residential Community Care Compound, 49% showed no evidence of DDIs. The remaining 51% had at least one potential DDI. In a total of these 807 patients, the mean age of the participants was 43.9 years with a standard deviation of 18.0. The majority fell within the 18 to 65 years age range, accounting for 81.7%. Around 44% of them had no comorbidities other than their psychiatric condition, and 56% had at least one or more medical condition. The most commonly documented psychiatric diagnoses were anxiety disorders (25.65%) and schizophrenia/schizoaffective disorders (23.9%), among a diverse range of mental illnesses. Table 2 illustrates the demographics and clinical characteristics.

### DDIs categories

On average, patients were taking 4.69 ± 3.36 medications, of which 2.23 ± 0.96 were psychotropics. From those who had a potential interaction, the average number of potential psychotropic drug interactions per patient was found to be 3.15. The total number of potential drug interactions detected in study participants is 2543 interactions. The most encountered DDIs were those that fall under Category C (86.20%; *n* = 2192), followed by Category D (7.86%; *n* = 200). Table 3 illustrates a description of potential DDIs.

### Critical DDIs identified

All of them are Pharmacodynamic Drug Interactions of types. The majority of these interactions were identified as potentially for developing QTc Prolongation/Torsade’s de Pointes (TdP) or anticholinergic intoxication.

Table 4 illustrates the description of Category X DDIs identified among these 807 patients.

### Association between demographic and treatment-related variables and level of severity (“Monitor/Low-Risk Interactions” vs. “Intervention Required/High-Risk Interactions”)

The result showed a significant difference in the total number of medications between the two groups being compared, as the *P*-value was equal to 0.00. However, there was no statistically significant difference in terms of main psychiatric diagnosis (*P* = 0.055), age (*P* = 0.365), gender (*P* = 0.794), number of comorbidities (*P* = 0.190), or level of prescriber (*P* = 0.187) as in Table 2.

### Predictors of potential “Intervention Required/High-Risk Drug Interactions”

Table 5 illustrates predictors of potential “Intervention Required/High-Risk” DDIs. The total number of medications was the only statistically significant predictor of having potential “Intervention Required/High-Risk Drug Interactions” (*P* < 0.001). For every additional medication, the odds of the outcome increase by approximately 14.9%.

## DISCUSSION

The findings from this study provide significant insights into the prevalence and characteristics of potential DDIs among patients receiving psychotropic medications at outpatient mental health services using the digital clinical decision support system Lexicomp^®^. There are a total of 23 publicly available applications that are used to check for potential DDIs.^23^ In HMC, the most common and widely used application for identifying potential DDIs is Lexicomp^®^ interact. The application combines all scientific information needed for understanding drug interactions in an electronic display website, providing an efficient way to inform health care professionals about adverse drug events that could affect patients’ care. It was found that, in comparison to Stockley’s Drug Interactions book, Lexicomp^®^ Interact has the best sensitivity and positive predictive value against potential DDIs.^24,25^ The application provides information on risk rating, severity, onset, and reliability rating in addition to patient management and further details discussion with evidence-based medicine resources.

Almost half of the sample exhibited at least one potential DDI, which aligns with existing literature indicating a high prevalence of potential DDIs in psychiatric populations. For instance, Aburamadan et al. studied the potential DDIs in psychiatric inpatients receiving psychotropic medications in the United Arab Emirates UAE reported that the overall frequency of potential DDIs among their study population was 64.7%.^26^ Santos et al. reported a wide range of clinically manifested potential DDIs, with prevalence rates varying significantly across studies, underscoring the importance of vigilant monitoring in clinical settings.^27^ The vast majority (86%) of the potential DDIs in our study fall under Category C, which recommends therapy monitoring, while Category D, which recommends therapy modification, represents around 8% and Category X, which recommends avoiding combination, is only identified in less than 1%. This highlights that the pharmacy services in the facility have made notable contributions by significantly reducing potential DDIs through effective pharmacy interventions and collaboration with the prescribing psychiatrists. The prevalence of Category D and X DDIs in our sample is much less when compared with the findings from Bačar Bole et al.,^28^ who examined potential DDIs in 311 admitted patients and reported that more than half of the patients had at least one Category X and about 77% of patients had at least one Category D DDI. However, the different settings and the wide range of benzodiazepine use, in addition to other psychotropics, in the inpatient setting, managing patients in the acute phase of their illness, might explain the difference in the results.

The average age of patients in our study was 43.9 years, which reflects demographic trends observed in similar studies where middle-aged adults are often the most frequent users of psychotropic medications.^29^

DDIs occur frequently, mainly affected by the patient’s age and the number of medications prescribed; the more the number, the higher the likelihood of them. In fact, DDIs risk can increase from 6% in patients taking two medications to 50% for those taking five medications, and can be 100% for those taking ten medications.^10^

Our data revealed that patients were prescribed an average of 4.69 medications, with 2.23 being psychotropic drugs. This level of polypharmacy is concerning, as it has been associated with an increased risk of adverse drug reactions and potential DDIs; however, it is still considered common clinical practice in managing patients with psychiatric conditions^29^. The total number of potential drug interactions detected was 2543, with an average of 3.15 interactions per patient. The analysis of our study revealed that the number of prescribed medications is the only statistically significant predictor of having potential “Intervention Required/High-Risk Drug Interactions,” and it was found that for every additional medication, the odds of the outcome increase by approximately 14.9%. As we have investigated the potential DDIs in outpatient mental health clinics, the number of interactions in relation to the medication used could be an implication of the polypharmacy of both psychotropic and non-psychotropic medications. The relation between polypharmacy and potential DDIs is identified in a study in the United Arab Emirates, which focused on potential DDIs among psychiatric inpatients receiving antipsychotic therapy, and found that polypharmacy was one of the significant predictors for potential DDIs.^26^ Patients with polypharmacy are at increased risk of getting drug interactions, as it was reported by Wolff et al. (OR, 3.7; 95% CI, 3.5–3.9) and Bačar Bole et al., who concluded that the number of prescribed drugs was a risk factor for potential DDIs (OR, 2.85; 95% CI, 1.84–5.73).^28,30^ Other demographic characteristics, such as age, gender, diagnosis, and comorbidities, were not statistically significant predictors of the potential DDIs.

The predominance of Category C interactions (86%) suggests that while these interactions may not require immediate modifications of therapy, they still necessitate careful monitoring and management.^31^ The identification of Category D interactions (7.8%) further emphasizes the need for clinicians to be aware of the potential for more severe consequences, as these interactions may require dose adjustments or alternative therapies.^32^ Marović et al. have studied the potential DDIs in outpatient settings and determined 2285 potential DDIs of which, 47.5% were psychotropic drug interactions. Among psychotropic drug interactions, 64.9% were Category C, 32.2% were Category D, and 2.9% were Category X DDIs. Benzodiazepines and Zolpidem were the most common medications involved in the DDIs.^33^

It is very important to identify the mechanism by which a given drug interaction occurs as this do impact the time course and the methods of avoiding the exact interaction. Occasionally, a potential DDI may be caused by two or more mechanisms simultaneously.^11^

Particularly alarming were the critical DDIs categorized as X, which were predominantly pharmacodynamic in nature, although they are less common. The majority of these interactions were associated with QTc prolongation and anticholinergic intoxication. Such findings are consistent with previous research that highlighted the dangers of combining certain psychotropic medications, particularly those that affect cardiac conduction or cholinergic pathways.^34^ The implications of these interactions are profound, as QTc prolongation can lead to life-threatening arrhythmias, necessitating proactive management strategies in clinical practice.^35^ Moreover, the study’s results resonate with the findings of Zapelini do Nascimento et al., who emphasized the need for continuous pharmacovigilance in the use of psychotropic drug combinations due to the high incidence of preventable interactions,^36^ which is already part of the practice of our pharmacists. The role of pharmacists in mitigating these risks cannot be missed and should be acknowledged, as demonstrated by AlRuthia et al., who found that pharmacist interventions significantly reduced the rate of potential DDIs among psychiatric patients.^37^ This also confirms that the interdisciplinary collaboration between prescribers and pharmacists within our facility plays a role in supporting patient safety in outpatient settings.

The clinical implications of potential DDIs in psychiatric patients are profound. Potential DDIs can lead to adverse drug reactions, which may exacerbate psychiatric symptoms or lead to physical comorbidities. Identifying high-risk drug combinations, such as those involving antipsychotics and benzodiazepines, can help prevent oversedation and respiratory depression when combined. The retrospective nature of many studies investigating potential DDIs poses challenges in assessing their clinical impact. For example, the risk of potential DDIs with antidepressants is often based on pharmacokinetic speculation; the actual clinical significance can vary widely among patients. This variability underscores the necessity for ongoing monitoring and individualized treatment plans to mitigate the risks associated with potential DDIs.

To our knowledge, this is the only study to evaluate significant DDIs among patients receiving psychotropic medications in Qatar. This gives the research a unique and relevant perspective for local clinical practice. With a sample size of 1586 patient files, we were able to gather substantial data to analyze the prevalence and patterns of these interactions. Most studies focus on inpatient care, where monitoring is more consistent, so our emphasis on outpatient care adds new insights into the risks in that setting, where monitoring may not be as frequent. Additionally, the fact that patients were prescribed an average of 4.69 medications raises concerns about polypharmacy, which increases the chances of potential DDIs and adverse reactions. The identification of critical interactions—like those leading to QTc prolongation and anticholinergic intoxication—further highlights the importance of careful management in these cases. Moreover, the real-time data entry, the use of standardized tools, and the clear inclusion and exclusion criteria reduced potential bias and heterogeneity, thereby limiting potential confounding.

However, there are some limitations. Since the study is retrospective, it relies on existing patient records, which means we were unable to directly assess the clinical outcomes or significance of the potential DDIs in each case. Additionally, we didn’t account for certain variables that could help identify which patients are more at risk, limiting the ability to suggest targeted interventions.

## CONCLUSIONS

In conclusion, the given prevalence of potential DDIs among patients receiving psychotropic medications underscores the need for continuous monitoring and management strategies. The findings of this study contribute to the growing body of literature advocating for enhanced pharmacovigilance and interdisciplinary approaches to medication management in psychiatric care. Future research should focus on identifying the possible variables associated with potential DDIs and developing and implementing effective strategies to minimize the risks associated with polypharmacy and potential DDIs, ultimately improving patient outcomes in mental health services.

## List of abbreviations

**Table:** 

CNS	central nervous system
DDD	defined daily dose
DDIs	drug-drug interactions
HMC	Hamad Medical Corporation
MHS	Mental Health Services
TdP	Torsades de Pointes
OR	odd ratio

## Ethical considerations

This work was approved by the Medical Research Center (MRC) at Hamad Medical Corporation (Grant number: MRC-01-22-773).

## Availability of data and materials

The datasets used and/or analyzed during the current study are available from the corresponding author on reasonable requests due to privacy and confidentiality considerations.

## Conflict of interest

All authors declare that they have no competing interests.

## Consent to participate declaration

This study was conducted retrospectively using data obtained from medical records. The need for informed consent was waived by the Institutional Review Board (IRB) due to the nature of the study. All procedures were in accordance with ethical standards, including those outlined in the Declaration of Helsinki.

## Authors’ contributions

Contribution to the idea conception, Contribution to study planning, design of the study: RA, OA. Data Collection: MA, SA, YE, NE, SH, RA. Data analysis, computation: OA. Manuscript writing: MA, RA, OA, YE, SA. Manuscript review and editing: RA, OA, MA, SA, YE, NE, SH, NA. Supervision of the project’s overall progress: RA, NE. All authors revised the final manuscript critically for intellectual content and gave their valuable feedback and final approval of this version to be published.

## Figures and Tables

**Table 1.** Lexicomp^®^ risk rating categories for drug, outcome severity, onset, and reliability rating.

Risk rating	Action
A	No interaction
B	No action needed
C	Monitor therapy
D	Modify regimen
X	Avoid combination
**Outcome severity**
Minor	No need for medical intervention
Moderate	Medical intervention is needed to treat the effects.
Major	Effects may result in death, hospitalization, permanent injury, or therapeutic failure.
**Onset of an unmanaged interaction**
Immediate	0–12 hours
Rapid	12–72 hours
Delayed	More than 72 hours
**Reliability rating**
Excellent	Multiple RCTs or a single RCT plus more than 2CR
Good	Single RCT plus fewer than 2 CR
Fair	>2 CR or <2 CR plus other supporting data
Poor	<2 case reports with no other supporting data

RCT: randomized controlled trials; CR: case reports.

**Table 2.** Patients’ demographics and clinical characteristics (N = 807).

Parameters	*n* (%)	Low-to-moderate risk (categories B and C)	High-risk (categories D and X)	P-value
Age, mean ± SD	43.86 ± 18.02	Mean rank 400.20	Mean rank 418.55	0.365*
Age category, *n* (%)				
<18	52 (6.44%)			
18–65	659 (81.66%)			
≥66	96 (11.9%)			
Gender, *n*(%)				**0.794^$^**
Female	421 (52.2%)	332 (41.1)	89 (11)	
Male	386 (47.8%)	308 (38.2)	78 (9.7)	
Number of comorbidities other than psychiatry			
None	355 (44%)			
1	137 (17%)			
2	107 (13.3%)			
3	97 (12%)			
4	50 (6.2%)			
5	33 (4.1%)			
6	16 (2%)			
>7	13 (1.6%)			
Comorbidities other than psychiatry (mean + SD)	1.65 + 1.85	Mean rank 395.27	Mean rank 420.46	0.190*
Mental illness diagnosis				**0.055^$^**
Schizophrenia spectrum and schizoaffective disorder	193 (23.9%)	138 (17.10)	55 (6.82)	
Bipolar affective disorder	98 (12.14%)	82 (10.16)	16 (1.98)	
Depression	177 (21.93%)	145 (17.97)	32 (3.97)	
Anxiety disorders	207 (25.65%)	170 (21.07)	37 (4.58)	
ADHD and LDs	37 (4.58%)	28 (3.47)	9 (1.12)	
Others (gender identity, personality disorder, suicidal thoughts, and others)	95 (11.77%)	77 (9.54)	18 (2.23)	
Level of prescribers				**0.187^$^**
Consultants	331 (41%)	272 (33.7)	59 (7.3)	
Fellows/specialists	367 (45.5%)	281 (34.8)	86 (10.7)	
Residents	109 (13.5%)	87 (10.8)	22 (2.7)	
**Total number of medications** (mean + SD)	4.69 ± 3.36	Mean rank 379.75	Mean rank 492.51	0.000*
**Number of psychotropics alone** (mean + SD)	2.23 ± 0.96	Mean rank 374.79	Mean rank 514.19	0.000*

Note: N represents the number of patients who had at least one DDI.

^$^Chi-square test.

*Mann-Whitney test.

**Table 3.** Drug-drug interactions identified (N = 2543).

Total number of interactions per patient (mean + SD)	4.46 ± 5.64
DDI category, *n* (%)	
Category B	136 (5.35%)
Category C	2192 (86.20%)
Category D	200 (7.86%)
Category X	15 (0.59%)

Note: N represents the total number of potential drug interactions detected in study participants.

**Table 4.** Category X drug-drug interaction identified (*n* = 15).

Interactions	Severity	Type of DDI	Onset	Reliability rating	Occurrence in *n* (%) of cases
**Quetiapine: Sulpiride**	Moderate	Pharmacodynamic Drug Interactions: QTc Prolongation and Torsades de Pointes (TdP)	Unknown	Fair	4 (0.6)
**Fluphenazine: Ipratropium**	Moderate	Pharmacodynamic Drug Interactions: Anticholinergic Intoxication	Unknown	Fair	2 (0.3)
**Nortriptyline: Ipratropium**	Moderate	Pharmacodynamic Drug Interactions: Anticholinergic Intoxication	Unknown	Fair	1 (0.1)
**Olanzapine: Cabergoline**	Moderate	Pharmacodynamic Drug Interactions: Diminish the antipsychotic effect	Unknown	Good	1 (0.1)
**Paliperidone: Cabergoline**	Moderate	Pharmacodynamic Drug Interactions: Diminish the antipsychotic effect	Unknown	Fair	1 (0.1)
**Flupentixol: Chlorpromazine**	Major	Pharmacodynamic Drug Interactions: QTc Prolongation and Torsades de Pointes (TdP)	Unknown	Fair	1 (0.1)
**Quetiapine: Chlorpromazine**	Major	Pharmacodynamic Drug Interactions: QTc Prolongation and Torsades de Pointes (TdP)	Unknown	Fair	1 (0.1)
**Flupentixol: Chlorpromazine**	Major	Pharmacodynamic Drug Interactions: QTc Prolongation and Torsades de Pointes (TdP)	Unknown	Fair	1 (0.1)
**Quetiapine: Methadone**	Major	Pharmacodynamic Drug Interactions: QTc Prolongation and Torsades de Pointes (TdP)	Unknown	Fair	1 (0.1)
**Fluphenazine: Orphenadrine**	Major	Pharmacodynamic Drug Interactions: enhance the CNS depressant effect of Orphenadrine	Unknown	Fair	1 (0.1)
**Paliperidone: Orphenadrine**	Major	Pharmacodynamic Drug Interactions: enhance the CNS depressant effect of Orphenadrine	Unknown	Fair	1 (0.1)

**Table 5.** Predictors of potential “Intervention Required/High-Risk Drug Interactions.”

	Significance	Exp(B)
Age	0.846	0.999
Gender	0.912	1.020
Level of prescribers	0.298	1.147
Number of comorbidities other than psychiatry	0.533	0.963
**Total number of medications**	**0.000**	**1.149**
Main psychiatric diagnosis	0.072	0.900
